# Comparative immunogenicity and structural analysis of epitopes of different bacterial L-asparaginases

**DOI:** 10.1186/s12885-016-2125-4

**Published:** 2016-02-11

**Authors:** Vadim S. Pokrovsky, Marat D. Kazanov, Ilya N. Dyakov, Marina V. Pokrovskaya, Svetlana S. Aleksandrova

**Affiliations:** V.N. Orekhovich Institute of Biomedical Chemistry, Moscow, Russia; N.N. Blokhin Cancer Research Center, Moscow, Russia; Research and Training Center on Bioinformatics, A.A. Kharkevich Institute for Information Transmission Problems, Russian Academy of Science, Moscow, Russia; I.I. Mechnikov Research Institute of Vaccine and Sera, Moscow, Russia

**Keywords:** L-asparaginase, Immunogenicity, Epitope

## Abstract

**Background:**

E.coli type II L-asparaginase is widely used for treatment of acute lymphoblastic leukemia. However, serious side effects such as allergic or hypersensitivity reactions are common for L-asparaginase treatment. Methods for minimizing immune response on L-asparaginase treatment in human include bioengeneering of less immunogenic version of the enzyme or utilizing the homologous enzymes of different origin. To rationalize these approaches we compared immunogenicity of L-asparaginases from five bacterial organisms and performed sequence-structure analysis of the presumable epitope regions.

**Methods:**

IgG and IgM immune response in C57B16 mice after immunization with Wollinella succinogenes type II (WsA), Yersinia pseudotuberculosis type II (YpA), Erwinia carotovora type II (EwA), and Rhodospirillum rubrum type I (RrA) and Escherichia coli type II (EcA) L-asparaginases was evaluated using standard ELISA method. The comparative bioinformatics analysis of structure and sequence of the bacterial L-asparaginases presumable epitope regions was performed.

**Results:**

We showed different immunogenic properties of five studied L-asparaginases and confirmed the possibility of replacement of EcA with L-asparaginase from different origin as a second-line treatment. Studied L-asparaginases might be placed in the following order based on the immunogenicity level: YpA > RrA, WsA ≥ EwA > EcA. Most significant cross-immunogenicity was shown between EcA and YpA. We propose that a long N-terminus of YpA enzyme enriched with charged aminoacids and tryptophan could be a reason of higher immunogenicity of YpA in comparison with other considered enzymes. Although the recognized structural and sequence differences in putative epitope regions among five considered L-asparaginases does not fully explain experimental observation of the immunogenicity of the enzymes, the performed analysis set the foundation for further research in this direction.

**Conclusions:**

The performed studies showed different immunogenic properties of L-asparaginases and confirmed the possibility of replacement of EcA with L-asparaginase from different origin. The preferable enzymes for the second line treatment are WsA, RrA, or EwA.

**Electronic supplementary material:**

The online version of this article (doi:10.1186/s12885-016-2125-4) contains supplementary material, which is available to authorized users.

## Background

L-asparaginase (EC 3.5.1.1.) *E. coli* type II (EcA) has been widely used for acute lymphoblastic leukemia treatment for more than 30 years. The mechanism of antileukemic activity is believed to be directly related to the hydrolysis of L-asparagine and subsequent significant depletion of L-asparagine concentration in blood and death of cells that are not able to express asparagine synthetase or have low level of expression [1]. It is known that hypersensitivity, including several allergic reactions and even anaphylactic shock, are among the most dangerous side effects of EcA treatment [2]. Moreover, even if a patient doesn’t have severe hypersensitivity symptoms, the development of anti-EcA antibodies could minimize the efficacy of treatment due to the alteration of pharmacokinetics and elimination the enzyme from the blood.

A number of approaches to prevent the immunogenicity of L-asparaginases has been investigated extensively: chemical modifications of epitopes to reduce the immunogenicity, site-directed mutagenesis of amino acid residues to diminish immunogenicity without reduction of enzymatic activity, consequent use of L-asparaginases with different immunogenic properties. The most common way is a chemical modification of the protein, for instance, conjugation with polyethylene glycol (PEG). It was shown that pegylation significantly increases the half-life (T_1/2_) of EcA in human serum, from 1.24 ± 0.17 to 5.73 ± 3.24 days [3, 4]. However, if the patient is allergic to native EcA, the T_1/2_ of pegylated EcA also decreases, which is due to identical antigenic epitopes [5]. Besides pegylation, several approaches have demonstrated the ability to decrease immunogenicity, namely encapsulation into liposomes 158–180 nm [6], immobilization into a biocompatible polyethylene glycol-albumin hydrogel [7], formulation of L-asparaginase load poly(lactide-to-glycolide) nanoparticles [8], chemical modification by N, O-carboxymethyl chitosan [9], etc. For example, encapsulation of palmitoyl-asparaginase into liposomes increases the T_1/2_ of native EcA for at least eight times [10]. Conjugation with low-immunogenic and non-toxic proteins, for instance silk fibroin, or encapsulation into red blood cells in vitro could be used for hiding the epitopes of L-asparaginases and, therefore, increase of T_1/2_ [11–14].

Epitope mapping and subsequent production of antigenically modified enzymes can be considered as the second efficient method to minimize immunogenicity. It has been proved that the major antigenic epitope of *Erwinia chrysanthemi* (ErA) is ^282^GIVPPDEELP^287^, and replacement Pro^285^ with Thr^285^ has led to 8-fold decrease of the immunogenicity of the native enzyme [15]. However, the immune response for large proteins is usually complex, and antibodies produced are usually polyclonal, hence the replacement of one amino acid can’t prevent the formation of antibodies against modified protein in hypersensitive mice previously treated with native one.

The third method that has been used is the consecutive administration of L-asparaginases with different antigenic properties. It is known that anti-EcA antibodies do not interfere with the ErA pharmacokinetics, which is the reason why ErA can be effectively used in patients previously treated with EcA [16–18]. Administration of new L-asparaginases, that do not have cross-reactivity with EcA and ErA, could be an effective approach for treatment of hypersensitive patients who have received multiple doses of EcA and/or ErA. It has been proven that *Helicobacter pylori* L-asparaginase has different immunogenic properties from EcA in mice [19]. During the last 7 years we’ve obtained and evaluated the enzymatic and anticancer properties of a few recombinant L-asparaginases from different origin, namely *Wollinella succinogenes* type II (WsA), *Yersinia pseudotuberculosis* type II (YpA), *Erwinia carotovora* type II (EwA), and *Rhodospirillum rubrum* type I [20–22].

The aims of this study were the evaluation of the immunogenicity in mice and cross-reactivity between these L-asparaginases and EcA, and elucidation of its structural basis based on analysis of three-dimensional structures.

## Methods

### Bioinformatics

Structure-based multiple sequence alignment of EcA, WsA, EwA, YpA and RrA was constructed using PROMALS3D [23]. Three-dimensional structure of the WsA, EwA, YpA and RrA proteins were modeled by I-TASSER [24]. Epitopes experimentally verified for ErA [15] were projected on the structure-based alignment using sequence-to-profile alignment method implemented in Clustal Omega [25]. Bioinformatics prediction of epitopes were made by Discotope [26], ElliPro [27] and EPSVR [28]. UCSF Chimera [29] was used for visualization of the 3D structure of enzymes. Solvent accessibility of the tetramer was calculated by DSSP [30].

### Reagents

L-asparagine (Reanal, Hungary); Na_2_HPO_4_, NaH_2_PO_4_, KCl (Serva, Germany); NaCl (Merck, Germany); Tween-20, NaHCO_3_, Na_2_CO_3_ (Sigma-Aldrich, USA). We used standard buffers: PBS, pH 7.4; carbonate-bicarbonate buffer 0.1 M, pH 9.5, citrate-phosphate buffer, 0.1 M, pH 5.0, PBS-Tween 0.05 %.

### Enzymes (antigens)

We used the commercially available lyophilized EcA preparation (Medak, Germany, 10 000 IU per vial); lyophilized recombinant EwA, that is similar to ErA (Additional file 1: Figure S1, http://purl.org/phylo/treebase/phylows/study/TB2:S18796) [22], lyophilized recombinant YpA [20], lyophilized recombinant RrA, obtained from IBMC RAMS [21], and lyophilized recombinant WsA, obtained from GosNIIgenetika.

### Animals

Female C_57_Bl_6j_ 8–12 weeks old mice were used for the in vivo studies. Mice were kept in animal facility of N.N. Blokhin Cancer Research Center of RAMS. All animal studies were carried out using procedures in compliance with EU (European Convention for the Protection of Animals Kept for Experimental and other Scientific Purposes, Strasbourg, 1985; 86/609/EEC and 2010/63/EU) directives on the protection of animals used for scientific purposes and according to institutional policy on the care and use of laboratory animals. The animal studies were approved by the local ethics committee of I.I. Mechnikov Institute of vaccine and sera, the decision from 26/01/2015.

### Immunogenicity studies

To evaluate the IgM immune response, groups of mice (five mice per group) received one i.v. injection of 500 μg of each preparation. To evaluate the IgG immune response 300 μg of each L-asparaginase were administered i.v. 3 times, at 2-week intervals. 0.9 % sodium chloride solution was injected in a separate groups of animals as controls (five mice per group). Blood samples were collected 7 days after the last immunization. Plasma samples for ELISA assay were obtained by centrifugation at 400 g at 4 °C for 10 min and stored at −80 °C until analysis. Then the samples were incubated for 10 min, centrifuged at 10,000 rpm (Eppendorf 1500) at 24 °C and were used immediately for experiments.

Serum Ab responses were determined using an ELISA kit (Maxisorb, Nunc). Briefly, standards, controls and pre-diluted samples of 100 μl of different L-asparaginases in carbonate-bicarbonate buffer, 5 μg/ml, were added into the wells of a 96-well plates and incubated at + 4 °C for 12 h. The plate was washed several times for 2 min with 300 μl phosphate buffer saline containing 0.05 % Tween 20 (PBS-Tween).

Serial dilutions (1:50 to 1:256,000) of mouse plasma were prepared. Following a 1 h incubation at 37 °C, the wells were washed as described above and the residual activity was measured. Serum Ab binding was detected using polyclonal secondary goat Ab to mouse total IgG or IgM. We used pre-diluted GoatAnti mouse IgG or GoatAnti mouse IgM, HU ADS biotin conjugate (Invitrogen, Cat# M30115) 1:10000, 100 μl in each well, and streptavidine conjugate of horseradish peroxidase STREPTAVIDIN HRP (AbD Serotec) 1:10000, according to manufacturer’s instructions. After suitable washing, standard buffer for ELISA was added and the plate was incubated for 15 min at room temperature. The reaction was stopped by adding 50 μm of 1.8 M H_2_SO_4_ before measuring the optical density at 450 nm using a plate reader Multiscan FC.

For IgG response the serum was considered negative (−) if geometric mean of the titers (GMT) was <50, positive: 51–600 (+), 601–30,000 (++), and >30,000 (+++). For IgM response the serum was considered negative (−) if GMT was <50, positive: 51–125 (+), 126–300 (++), and >300 (+++).

### Statistical analysis

Geometric mean of the titers (GMTs) and the GMT ratios with corresponding 95 % confidence intervals (CIs) were calculated by taking the antilog of the mean of the log_е_-transformed data (assuming that log_е_-transformation of the titers follows a normal distribution).

To compare immunogenicity of different antigens we used GMT of each antigen reacting with the serum sample from mice immunized with the same antigen. For instance, EcA + anti-EcA vs YpA + anti-YpA. To prove statistical validity of cross-immunogenicity between groups we used GMT of each antigen reacting with the serum sample from mice immunized with the different antigen vs antigen reacting with the serum sample from control mice (no immunization).

SPSS 21 software was used for statistical analysis. One-way ANOVA was used to compare immunogenicity of antigens. Post hoc Dunnett’s T3 test was performed to assess differences between the individual groups. Calculations began with the logarithmic transformation of the antibody titers. *P* value <0.05 was considered statistically significant.

## Results

### IgG immune response

**Fig. 1:**
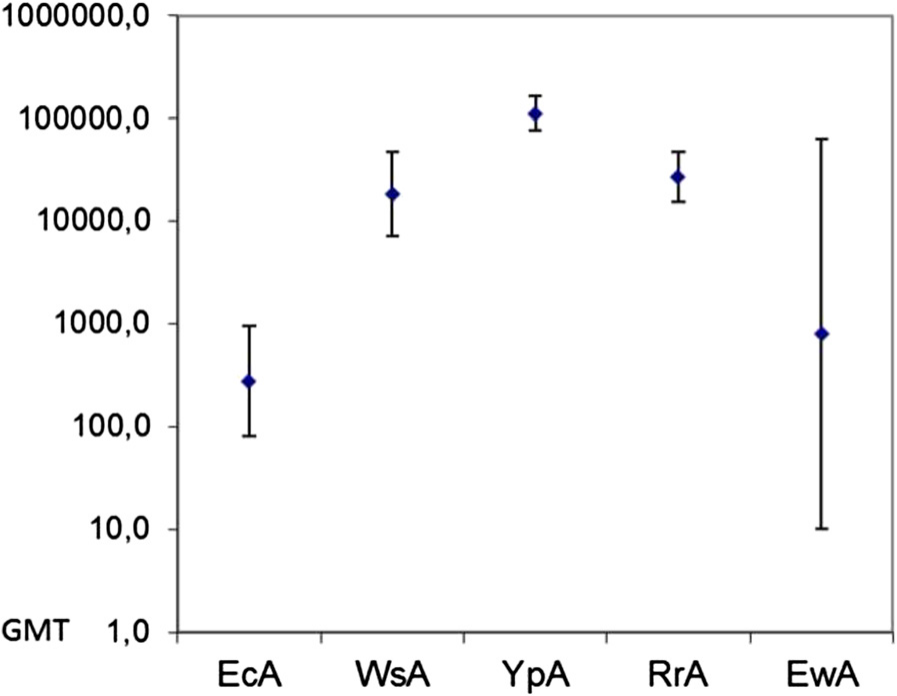
GMTs for serum anti-L-asparaginases-specific neutralizing antibodies, IgG response

The anti-L-asparaginase IgG were developed following repeated i.v. administration of enzymes. The most immunogenic L-asparaginase was found to be YpA (Table 1). GMTs of different enzymes varied from 275 (95 % CI 80–949) for EcA to 111431 (95 % CI 75831–163743) for YpA, with 795 (95 % CI: 10–63293) for EwA, 18379 (95 % CI: 7159–47182) for WsA and 26909 (95 % CI: 7159–47182) for RrA. The difference between YpA and all other enzymes was statistically significant (*p* < 0.001), suggesting YpA was most immunogenic compared to EcA, EwA, WsA and RrA in murine model. WsA, RrA and EwA showed similar immunogenicity without statistically significant differences. Thus, studied L-asparaginases might be placed in the following order based on the immunogenicity: YpA > RrA, WsA ≥ EwA > EcA (Fig. 1, Table 2).

**Table 1:** Immunogenicity of L-asparaginases, IgG response

Antigen for immunization	Samples for reaction				
	EcA	WsA	YpA	RrA	EwA
Control	–	–	–	–	–
EcA	+	–	–	–	–
WsA	+	++	–	–	–
YpA	++	+	+++	+	–
RrA	–	–	+	++	–
EwA	+	–	–	–	++

**Table 2:** Pair-wise comparison of immunogenicity of L-asparaginases, IgG response, post hoc Dunnett’s T3 test

Antigen for immunization	p			
	WsA	YpA	RrA	EwA
EcA	0.001	<0.001	0.001	0.989
WsA		0.026	0.954	0.440
YpA			0.005	0.169
RrA				0.352

### IgM immune response

The inhibitory titers per mice were the following: 1:400–1:1600 for YpA, 1:400–1:800 for RrA and 1:200–1:800 for EcA, EwA and WsA, GMTs are 400 (95 % CI: 218–735) for EcA, 528 (95 % CI: 244–1140) for WsA, 210 (95 % CI: 5–9036) for YpA, 159 (95 % CI: 5–5520) for RrA, 459 (95 % CI: 224–944) for EwA (Table 3). All enzymes showed similar immunogenicity without statistically significant differences.

**Table 3:** Immunogenicity of L-asparaginases, IgM response

Antigen for immunization	Samples for reaction				
	EcA	WsA	YpA	RrA	EwA
Control	–	–	–	–	–
EcA	+++	+	+	–	+
WsA	+	+++	+	+	+
YpA	+	++	++	–	+
RrA	–	+	+	++	+
EwA	–	+	+	+	+++

### Cross-reactivity of L-asparaginases

As it is shown in Table 4, sera from mice injected with EcA 3 times showed no statistically significant cross-reactions with all other enzymes. The immunization with WsA led to formation of antibodies against EcA (*p* = 0.029 compared with naïve control), however, the GMT was 353 times (WsA/EcA × 353) lower than for GMT for WsA + anti-WsA reaction. Sera obtained from mice treated with YpA were positive to all other enzymes if compared with naïve control with the following ratio ratings: YpA/EcA × 169, YpA/WsA × 308, YpA/RrA × 671 and YpA/EwA × 2228, displaying EcA as an antigenically closest equivalent to YpA. After multiple administration of RrA the statistically significant immune response was detected against YpA: RrA/YpA × 215. For EwA: EwA/EcA × 5, suggesting that the strongest immune response among studied enzymes can be obtained in mice treated with EcA after EwA.

**Table 4:** The cross-neutralization pattern of immunized mice sera after different L-asparaginase administration, IgG-response

Antigen for immunization	GMT/p	GMT, 95 % CI				
		EcA	WsA	YpA	RrA	EwA
EcA	GMT		2.2 (0.2–19.2)	2.6 (0.2–38.4)	6.9 (0.3–184.0)	6.6 (0.2–176.2)
	p		0.935	0.875	0.413	0.432
WsA	GMT	52.3 (2.8–979.2)		6.9 (0.3–184.0)	5.7 (0.3–115.3)	10.5 (0.7–149.6)
	p	0.029		0.437	0.521	0.277
YpA	GMT	659.8 (178.9–2433.6)	362.4 (77.0–1705.7)		165.7 (3.3–8296.3)	50.0 (50.0–50.0)
	p	<0.001	<0.001		<0.001	0.003
RrA	GMT	4.7 (0.0–663.9)	No response	125.0 (125.0–125.0)		7.1 (0.2–257.2)
	p	0.475		0.002		0.284
EwA	GMT	148.7 (85.6–258.0)	No response	7.1 (0.2–257.2)	8.9 (0.2–514.8)	
	p	<0.001		0.201	0.135	

Evaluation of IgM immune response confirmed the cross-reactivity between EcA and YpA. The most significant cross-reactions were: GMT for EcA: EcA/YpA × 4.6; GMT for WsA: WsA/EwA × 4.6 and WsA/RrA × 4.6; GMT for YpA: YpA/WsA × 1.6 and YpA/EcA × 2.8; GMT for RrA: RrA/YpA × 1.5; GMT for EwA: EwA/YpA × 4.6. All cross-reactions were statistically significant against naïve control (*p* <0.001). However, the biomedical implications of these findings can be compromised due to very low intrinsic IgM-response of all studied proteins (see section “IgM immune response”) and presumable non-specific reactions of serum in sensitized animals.

### Prediction and structural comparative analysis of epitopes

**Fig. 2:**
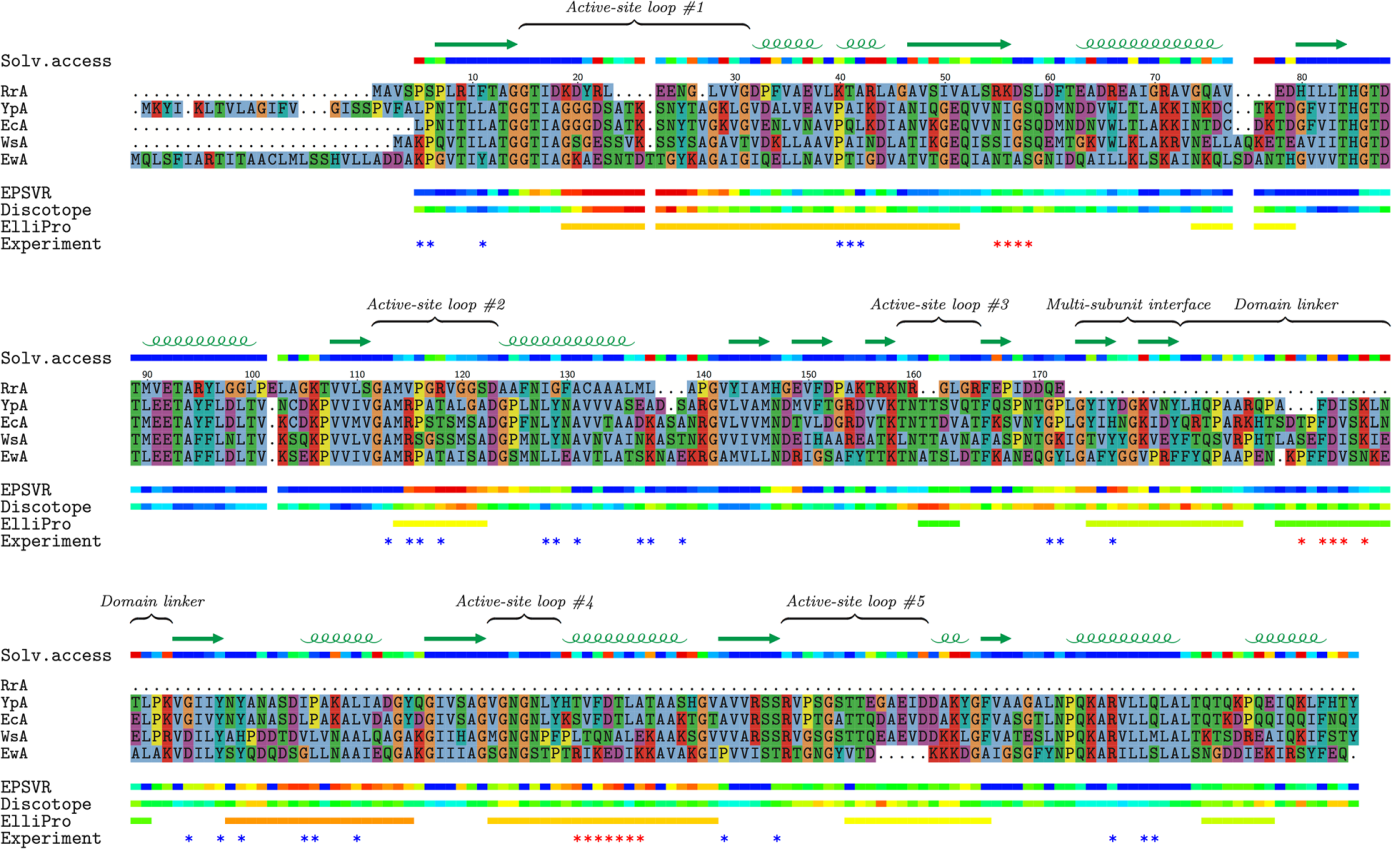
Structure-based multiple sequence alignment of five studied L-asparaginases. Four lines below the alignment show putative epitope region predicted by EPSVR, Discotope, and ElliPro methods, respectively, as well as the experimentally known epitopes projected from the *Erwinia chrysanthemi* L-asparaginase. Lines above the alignment represent the secondary structure and the estimation of solvent accessibility of EcA tetramer

To elucidate a nature of the L-asparaginase immunogenicity and to optimize the development of the bacterial L-asparaginases with reduced immunogenicity for therapeutic use, we performed a comparative structural and sequence analysis of putative epitopes. The presumable epitope regions were obtained using bioinformatics prediction and sequence-based projection of the experimental data existing for homologous proteins. First, we mapped experimentally verified epitopes known for ErA, which is the close homolog of the five considered enzymes, into 3D structure of enzymes using multiple sequence alignment. A solved three-dimensional structure of the EcA enzyme was taken from PDB, whereas 3D structures of other four enzymes were modeled using state-of-the-art software (see Methods) implementing homology modeling approach. Second, we used three bioinformatics methods to obtain consistent protein epitope predictions, which support and complement the experimental epitope projection results. The results of these methods consistently indicated nine regions of the enzyme as putative epitopes (Fig. 2).

**Fig. 3:**
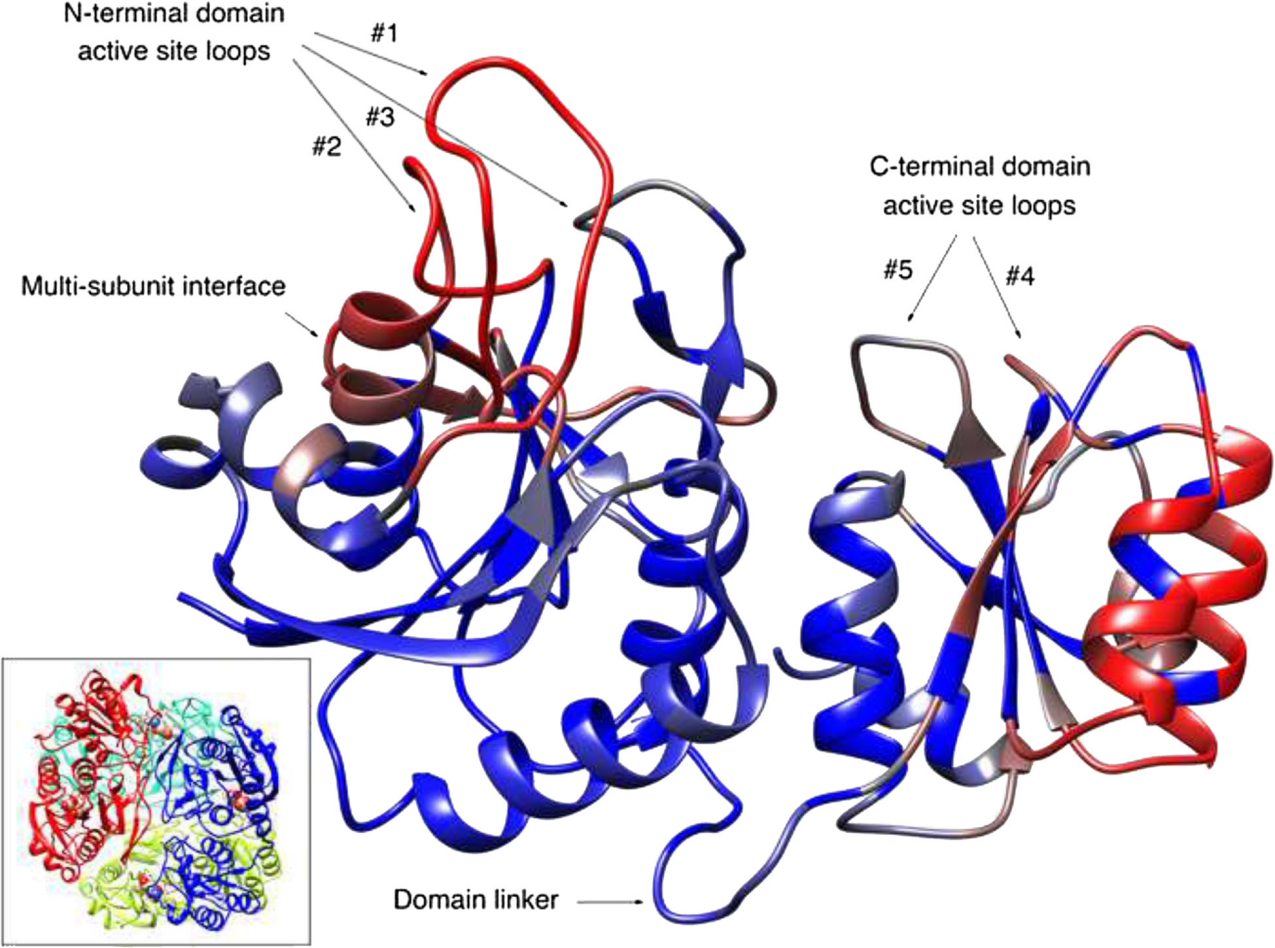
Three-dimensional structure of the EcA protein with a color mapping representing the example of the epitope prediction obtained by EPSRV method (blue – low probability, red – high probability). Following key structural elements of the enzyme are designated in the figure: three N-terminal active site loops, two C-terminal active site loops, multi-subunit interface regions and inter-domain linker. *Inset*: Tetrameric structure of *Escherichia coli* L-asparaginase EcA

Five out of these nine regions represent active site loops (Fig. 3), three of which are located in the N-terminal domain and two in the C-terminal domain. Four other putative epitope regions are two C-terminal domain helices, a region consisting of two parallel beta-strands involved in inter-subunit contacts and inter-domain linker. Since the bioinformatics methods of epitope prediction were applied for monomeric protein, we additionally investigated accessibility to solvent all of the predicted regions as a part of tetrameric structure to check whether these regions are accessible to antibodies (Fig. 2). Among the projected experimentally verified epitopes, two most confident ones are the regions of the active site, of which the first is located in the longest and highly protruding N-terminal loop (active site loop#1 in the Fig. 3) and the second is located in the C-terminal helix near the loop participating in the active site contacts with the substrate [31].

**Fig. 4:**
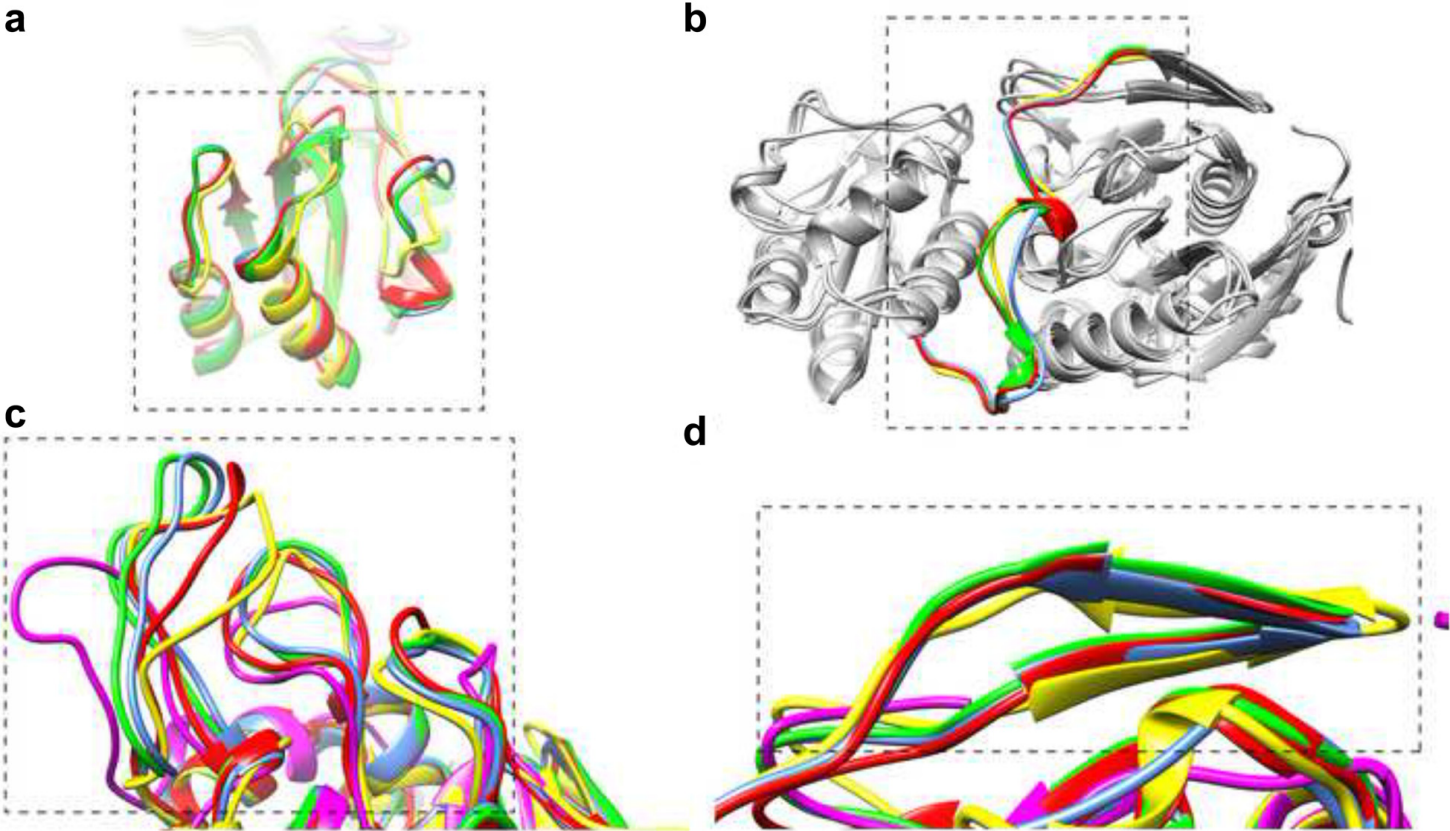
Superimposition of the predicted epitopes of EcA (*red*), EwA (*yellow*), WsA (*green*), YpA (*blue*) L-asparaginases including C-terminal active site loops (**a**), inter-domain linker (**b**), N-terminal active site loops (**c**), and multi-subunit interface regions (**d**)

We performed structural comparison of the putative epitope regions for all five studied L-asparaginases. Superimposition of the N-terminal domain parts of the active site for five considered proteins did not show any substantial structural differences for the three active site loops (Fig. 4c). The only detectable difference worth mentioning is the shorter length of the RrA loops. Predicted epitopes located in loops and helices of the C-terminal part of the active site of four enzymes except RrA (due to lack of C-terminal domain) are also structurally very similar to each other (Fig. 4a), except for a much shorter active site loop (active site loop#5 in the Fig. 3) of the EwA. Two antiparallel beta-strands of the multi-subunit interface show almost complete similarity in structural sense (Fig. 4d). Some structural differences were detectable among the domain linkers (Fig. 4b). Thus, the YpA enzyme has the shortest domain linker in comparison to other three enzymes, EcA and WsA enzymes have the longest ones, and EwA linker has an intermediate length.

We also analyzed amino acid content of the presumable epitope regions in order to compare enrichment in amino acids, which are known for increased immunogenicity — tyrosine, tryptophane and charged aminoacids [32]. The N-terminal active site loops of the four type II asparaginases have sufficiently similar amino acid content, whereas the RrA has a larger number of charged amino acids. Putative C-domain epitope regions are sufficiently conserved among studied L-asparaginases, although the EwA has a larger number of charged amino acids. A multi-subunit interface represented by two short antiparallel strands contains three tyrosines and two charged amino acids in case of YpA and WsA, two tyrosines and two charged amino acids in EcA and one tyrosine and one charged amino acid in EwA. Amino acid content of the inter-domain linker region does not show any substantial differences among the considered enzymes.

## Discussion

We performed a comparative analysis of structure and sequence of the bacterial L-asparaginase epitope regions, which were predicted by bioinformatics methods and projected from the experimentally known epitope regions of the homologous proteins. Particularly, we analyzed whether the presumable epitope regions differ among considered enzymes and whether these differences are associated with known structure and sequence elements that influence immunogenicity. Analysis of the secondary structure showed that the secondary structure is highly conserved among considered enzymes and most of the presumable epitopes regions are located in loops that is in accordance with structural studies [32]. Protrusion and solvent accessibility of the considered loops – two properties that are known for affecting immunogenicity – are sufficiently similar among the enzymes, except for N-terminal active site loops of the RrA, which are much shorter than the same loops of other enzymes. We also found a moderate differences among enzymes in a length of the inter-domain linker region.

Analysis of differences in amino acids associated with increased immunogenicity – tyrosine, tryptophane and charged amino acids – showed more differences than the structural analysis. Among these differences the most correlated with experimental data is the difference among enzymes in enrichment of tyrosines and charged amino acids in the multi-subunit interface region. However, structural properties of this region – a presence of two beta-strands and a moderate solvent accessibility – is not typical for epitopes. Apart from the predicted epitopes the N- and C-terminal ends of protein could be also considered as putative epitopes when they are long enough to possess enough protrusion and flexibility – two characteristic epitope properties. We speculate that a long N-terminus of YpA enzyme (see sequence alignment in Fig. 2), which is also enriched at the beginning with charged aminoacids and tyrosine (KYIK), could be a reason of higher immunogenicity of YpA in comparison with other considered enzymes. Overall, although the recognized structural and sequence differences in putative epitope regions among five considered L-asparaginases do not fully explain experimental observation of the immunogenicity of the enzymes, the performed analysis set the foundation for further research in this direction when more data are available.

There are two approaches to describe the cross-reactivity of antibodies: either L-asparaginase could be considered as an antigen for immunization or the enzyme is defined as a target for antibodies. The obtained data showed that after single and threefold administration all investigated L-asparaginases displayed moderate immunogenicity with low cross-reactivity. The antibodies produced in mice after EcA administration have the worst capacity for development of cross-reactivity with other enzymes. This phenomenon could be explained mainly by low immunogenicity of EcA, confirming the current position of EcA as a first-line L-asparaginase for acute lymphoblastic leukemia treatment. Any other enzyme, preferably WsA, YpA or RrA could be used as a second-line treatment without a risk of significant alteration of pharmacokinetics due to antibodies formation. However, EcA could be a target for antibodies produced in mice previously immunized with any other L-asparaginase. It could be explained both by higher immunogenicity of these enzymes and presence of similar epitopes in EcA and other enzymes, that was shown by analyses of three-dimensional structures. The affinity of cross-binding with EcA and other L-asparaginases vary directly as the average immunogenicity of each enzyme: YpA showed the highest cross-reactivity, that can be explained by more than 74 % homology of the sequences of EcA and YpA. The differences in cross-reactivity of each L-asparaginase evaluated by these two different approaches could be explained by different location of antigenic epitopes (internal or external epitopes).

The clinical relevance of our findings can be evaluated after comprehensive preclinical investigation of safety and efficacy of obtained enzymes and consequent clinical trials. The clinical pathway requires comparative trials of efficacy and safety of new L-asparaginases in patients with acute lymphoblastic leukemia, previously treated with EcA.

## Conclusions

The performed study showed different immunogenic properties of L-asparaginases and confirmed the possibility of replacement of EcA with L-asparaginase from different origin. YpA was most immunogenic enzyme compared to EcA, EwA, WsA and RrA in murine model. Based on these data, the preferable enzyme for the second line treatment is WsA, RrA or EwA. Further investigations of the epitopes and immunogenicity of novel L-asparaginases are needed to assume their therapeutic applications or create the mutated proteins with minimized immunogenicity in order to increase the efficacy of leukemia treatment.

## Additional file

Additional file 1: Figure S1. Phylogenetic tree of the WaA, YpA, EwA, RrA, EcA, ErA L-asparaginases. ErA has a minimal evolutionary distance to the EwA L-asparaginase. (PNG 20 kb)

## Abbreviations

EcA: *Escherichia coli* L-asparaginase
WsA: *Wollinella succinogenes* L-asparaginase
EwA: *Erwinia carotovora* L-asparaginase
YpA: *Yersinia pseudotuberculosis* L-asparaginase
RrA: *Rhodospirillum rubrum* L-asparaginase
PEG: polyethylene glycol
IgG: immunoglobulin G
IgM: immunoglobulin M
ELISA: enzyme-linked immunosorbent assay
