# Supplementary figures and images for: Comparative immunogenicity and structural analysis of epitopes of different bacterial L-asparaginases
Source: BMC Cancer. 2016 Feb 11;16:89. doi: 10.1186/s12885-016-2125-4 (PMC4750198; doi:10.1186/s12885-016-2125-4)

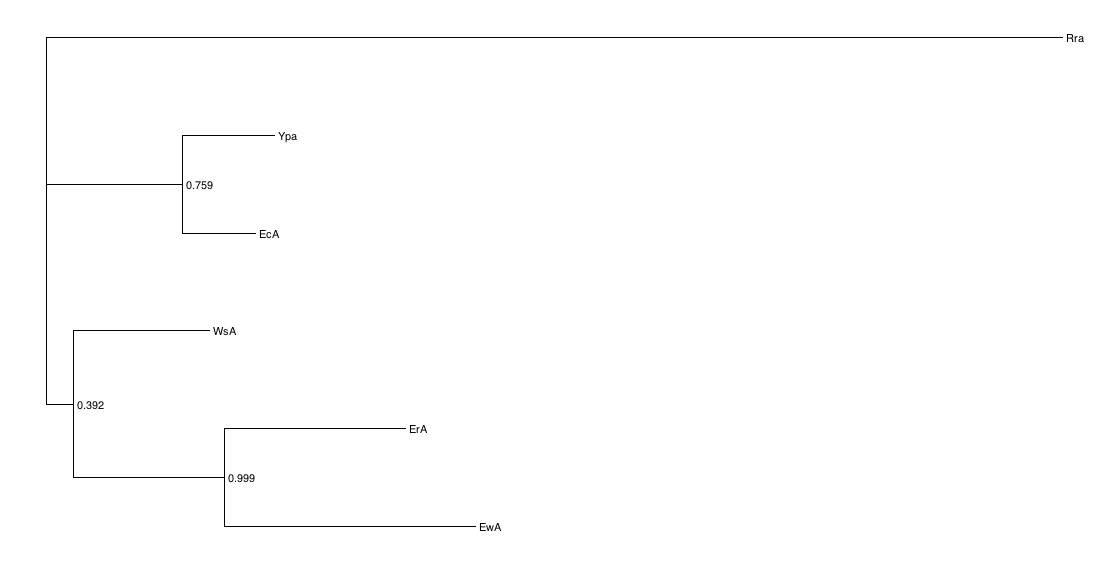

Supplement: Additional file 1: Figure S1. — Phylogenetic tree of the WaA, YpA, EwA, RrA, EcA, ErA L-asparaginases. ErA has a minimal evolutionary distance to the EwA L-asparaginase. (PNG 20 kb) [file 12885_2016_2125_MOESM1_ESM.png]
